# Spatially Confined Spin Polarization and magnetic sublattice control in (La,Sr)MnO_3−δ_ Thin Films by Oxygen Vacancy Ordering

**DOI:** 10.1038/s41598-017-04103-y

**Published:** 2017-06-29

**Authors:** Magnus Moreau, Sverre M. Selbach, Thomas Tybell

**Affiliations:** 1 Department of Electronic Systems, NTNU - Norwegian University of Science and Technology, 7491 Trondheim, Norway; 2 Department of Materials Science and Engineering, NTNU - Norwegian University of Science and Technology, 7491 Trondheim, Norway

## Abstract

Perovskite oxides are known for their strong structure property coupling and functional properties such as ferromagntism, ferroelectricity and high temperature superconductivity. While the effect of ordered cation vacancies on functional properties have been much studied, the possibility of tuning the functionality through anion vacancy ordering has received much less attention. Oxygen vacancies in ferromagnetic La_0.7_Sr_0.3_MnO_3−δ_ thin films have recently been shown to accumulate close to interfaces and form a brownmillerite structure (ABO_2.5_). This structure has alternating oxygen octahedral and tetrahedral layers along the stacking direction, making it a basis for a family of ordered anion defect controlled materials. We use density functional theory to study how structure and properties depend on oxygen stoichiometry, relying on a block-by-block approach by including additional octahedral layers in-between each tetrahedral layer. It is found that the magnetic and electronic structures follow the layers enforced by the ordered oxygen vacancies. This results in spatially confined electronic conduction in the octahedral layers, and decoupling of the magnetic sub-lattices in the octahedral and tetrahedral layers. These results demonstrate that anion defect engineering is a promising tool to tune the properties of functional oxides, adding a new avenue for developing functional oxide device technology.

## Introduction

The ABO_3_ perovskite structure is prone to changes in stoichiometry, and recently there has been considerable interest in layered perovskite-derived structures like the Ruddlesden-Popper, Aurivillius and Dion-Jacobsen families with cation ordering^1–3^. Layering enforces anisotropic properties in the materials by decoupling the octahedral building blocks, and enabling functional properties such as ferroelectricity^3^, ferromagnetism^4^, and superconductivity^5^, making them interesting for novel device applications such as tunable microwave filters and optoelectronic components^1, 6^. Interfaces between, or superlattices of, perovskites has also been used to confine electrons into 2 dimensions resulting in a 2-dimensional electron gas, where the most studied system is the LaAlO_3_/SrTiO_3_ (LAO/STO) interface^7–9^. Combining spin polarization with 2-dimensional conductivity is interesting both on a fundamental level as well as for spintronic applications^10^. Such realizations have been achieved in various superlattice configurations such as LaMnO_3_/SrMnO_3_^11–13^, LaAlO_3_/SrMnO_3_^14^, and SrTiO_3_/SrRuO_3_^15^.

While most of these studies have been on the ordering of cations and cation vacancies, anion vacancy ordering has recently been demonstrated as a route for altering the properties of thin films^16, 17^. Further it has been shown experimentally that it is possible to order anion vacancies in layers in thin films of ferromagnetic (La,Sr)MnO_3−δ_ (LSMO), *e*.*g*. in a brownmillerite structure with ABO_2.5_ stoichiometry^18–20^, making LSMO a model system for controlling functionality through anion ordering.

**Figure 1:**
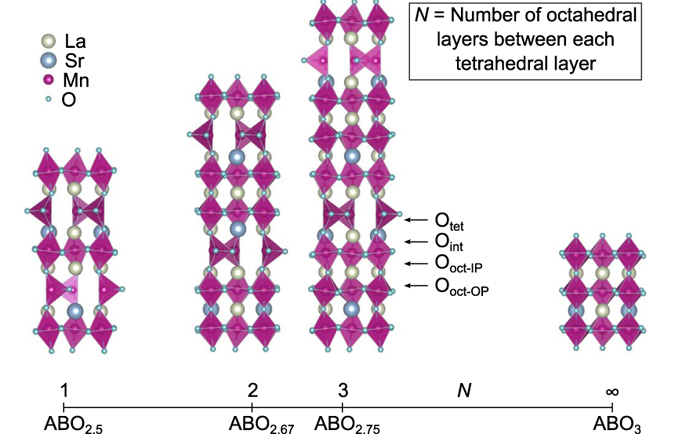
The evolution from brownmillerite, ABO_2.5_, to perovskite, ABO_3_ for (La,Sr)MnO_3−δ_ (LSMO). The labels O_tet_, O_int_, O_oct-IP_ and O_oct-OP_ defines the different oxygen positions. Note that there is no O_oct-OP_ for ABO_2.5_ and no O_tet_ and no O_int_ for ABO_3_.

Inspired by the possibility to realize layered oxygen vacancy ordered thin films with different stoichiometry^21^, we investigate oxygen vacancy ordering as a tool for controlling the magnetic properties and electronic structure of oxygen deficient LSMO. The brownmillerite structure consists of alternating BO_6_ octahedral layers and BO_4_ tetrahedral layers along the stacking direction. An increasing number octahedral layers between the oxygen deficient tetrahedral layers is schematically shown in Fig. 1, where the oxygen stoichiometry can be controlled in a block-by-block approach, going from ABO_2.5_ with one octahedral and one tetrahedral layer superlattice (1:1), *via* ABO_2.67_ and ABO_2.75_ for 2:1 and 3:1 superlattices, respectively, approaching ABO_3.0_ for a pure octahedral based thin film^22^. We find that the ordering of oxygen vacancies decouples the magnetic sublattices in the octahedral and tetrahedral layers. Furthermore, a large electronic band gap and low dispersion in the tetrahedral layers results in electron confinement in the octahedral layers.

## Results and Discussion

### Atomic structure

Before relying on a block-by-block approach to investigate the magnetism, we start by defining the stoichiometric ABO_3.0_ system and the (1:1) ABO_2.5_ oxygen deficient system. We lock the in-plane lattice parameters to that of STO, in order to simulate the effect of the substrate on the thin film. For the La_0.75_Sr_0.25_MnO_3_ system we reproduce the experimentally known ferromagnetic ground state^23^, where the energy difference compared to the most stable antiferromagntic ordering is 42.3 meV per formula unit (f.u.) as shown in Table 1. For the ABO_2.5_ system different oxygen vacancy positions within the unit cell were investigated, both oxygen vacancy ordered tetrahedral layers, as well as disordered vacancies throughout the unit cell^18^. The lowest energy is found for the experimentally reported^18–20^ antiparallel alignment of the two tetrahedrons as shown in Fig. 1. The ordered oxygen vacancies make up tetrahedral chains that can have either left- of right-handed rotations. There are three symmetry equivalent combinations of these tetrahedral rotation modes, a left-left left-left (LL-LL) with a *I*2*bm*-like symmetry, right-left left-right RL-LR with *Pbcm*-like symmetry, or left-left right-right (LL-RR) with *Pnma*-like symmetry^24^. For the ABO_2.5_ system strained to STO we find that the LR-RL tetrahedral chain with *Pbcm*-like symmetry is always lowest in energy for any given magnetic ordering. Due to the discrete Sr doping the structural models used in the calculations have *P*1 symmetry. When investigating the Mn-O bond lengths shown in Table 2, the Mn_oct_-O_int_ bond is found to be considerably longer than the other bonds for all values of *N*, the number of octahedral layers in-between each tetrahedral layer. However, the Mn_oct_-O_int_ bond does not follow a monotonic decreasing trend with increasing oxygen content. As shown in Table 2, it is longer for *N* = 1 and *N* = 3 (ABO_2.5_ and ABO_2.75_) than it is for *N* = 2 (ABO_2.67_). This is a well-known phenomenon for other layered perovskite system^25^, and is related to the different symmetries exhibited by odd and even numbers of *N*, giving rise to different octahedral and tetrahedral rotations. To test this scenario the Mn_oct_-O_int_-Mn_tet_ angle, the tetrahedral chain distance *R*, and octahedral bond length variation Δ as defined by Young and Rondinelli^24^ was also calculated. As shown in Table 2, the data with deviating Mn_oct_-O_int_-Mn_tet_ angle and *R* value for ABO_2.67_ points towards a symmetry induced rotation difference. Still, the longer Mn_oct_-O_int_ bond length can reduce the coupling between octahedral and tetrahedral layers^1^, and thus enable different magnetic sublattices in these layers.

**Table 1:** Energy differences of different spin orderings for stoichiometric ABO_3_ system.

Spin ordering:	G-type	FM	C-type	A-type
ΔE [meV/f.u.]	87.1	0	52.5	42.2

**Table 2:** Average Mn-O bond lengths and Mn-O-Mn angles as a function of oxygen stoichiometry, as well as *R* and Δ.

	ABO_2.5_	ABO_2.67_	ABO_2.75_	ABO_3_
Mn_oct_ - O_oct-IP_ [Å]	1.958	1.955	1.962	1.962
Mn_oct_ - O_oct-OP_ [Å]	N/A	1.998	1.985	1.957
Mn_oct_ - O_int_ [Å]	2.247	2.159	2.191	N/A
Mn_tet_ - O_tet_ [Å]	2.073	2.069	2.065	N/A
Mn_tet_ - O_int_ [Å]	1.989	2.000	1.996	N/A
∠ Mn_tet_ - O_int_ - Mn_oct_ [°]	153.13	157.18	154.95	N/A
*R* [Å]	4.899	4.923	4.909	N/A
Δ [×10^4^]	50.639	15.231	13.820	0.9622

The subscripts are defined in Fig. 1.

### Magnetic structure

#### ABO_2.5_

**Figure 2:**
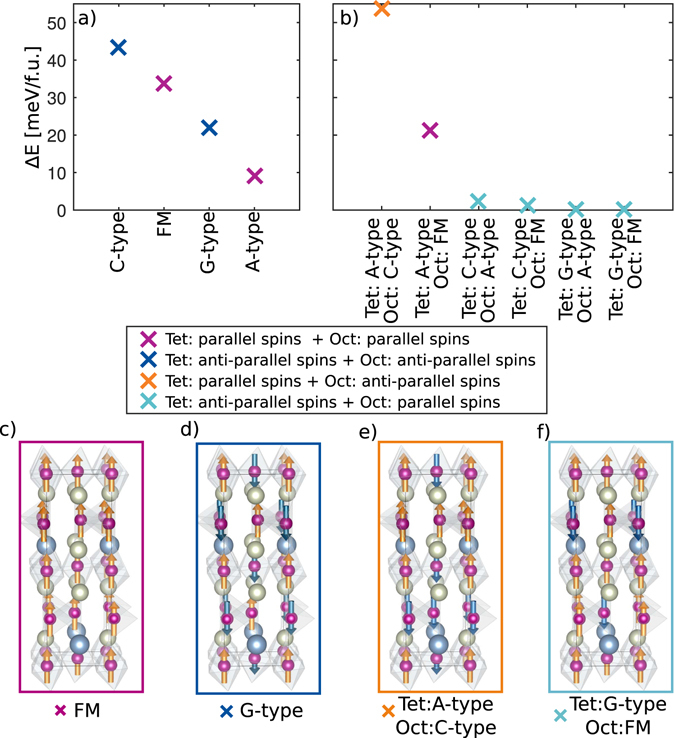
(**a**,**b**) Energy difference between different magnetic structures in the LSMO brownmillerite cell. (**a**) Same spin ordering in the entire structure. (**b**) Different magnetic sublattices in the tetrahedral (Tet) and octahedral (Oct) layers. It is possible to order the magnetic sublattices in four categories, in-plane parallel spins in both octahedral and tetrahedral layers, in-plane anti-parallel spins in both the octahedral and tetrahedral layers, and in-plane parallel spins in only the tetrahedral or only the octahedral layers. The lowest energy is observed when the octahedral layers have in-plane parallel spins and the tetrahedral layers have in-plane anti-parallel spins. (**c**–**f**) Depicts one example of spin ordering corresponding to each of the four categories.

Next the magnetic ground state of the ABO_2.5_ system is established. First it is assumed that the unit cell has a given magnetic order throughout the unit cell, either, ferromagnetic (FM), A-, C-or G-type antiferromagnetic (AFM) order. The total energy for the different spin orderings are shown in (Fig. 2a), revealing that the ABO_2.5_ system is prone to A-type AFM ordering. This differs from other brownmillerite oxides, *e*.*g*. SrCoO_2.5_ which was found to have a G-type AFM ground state^26^.

A-type spin ordering in ABO_2.5_ implies anti-parallel spins in the octahedral and tetrahedral layers, in agreement with the possibility of different magnetic sublattices in the octahedral and tetrahedral layers. Motivated by this idea, and that the longer Mn_oct_-O_int_ can decouple the magnetic sublattices, we investigate the effect of different magnetic sublattices in the various layers. Different magnetic sublattices have also earlier been suggested to explain an anomaly in the magnetic susceptibility of brownmillerite CaFeO_2.5_^27, 28^. Parallel ordering of spins in the octahedral layers combined with anti-parallel ordering of spins in the tetrahedral layers are found to be more stable than other configurations with parallel spins in the tetrahedral layers and/or anti-parallel spins octahedral layers, as shown in (Fig. 2b). Hence, by enforcing a parallel spin ordering in the octahedral layers and anti-parallel spins in the tetrahedral layers we get four possible combinations of spin ordering in the investigated brownmillerite unit cell (Fig. 2). For the octahedral layers there are two possibilities; firstly, all octahedral layers can be parallel, giving a net moment to the unit cell; or secondly, every second octahedral layer can be anti-parallel in an A-type like AFM ordering between the octahedral layers, resulting in no net moment. There are also two possibilities in the tetrahedral layers, C- and G-type AFM ordering. For C-type AFM ordering each tetrahedral layer has the same configuration, while for G-type AFM every second tetrahedral layer have opposite spin directions. Note that an A-type like order in the tetrahedral layers is not favored as A-type order has parallel spins within one tetrahedral layer. As shown in (Fig. 2b), the energy differences between these four spin orderings are small, less than 1 meV/f.u., and thus in the order of the resolution limit of density functional theory (DFT) calculations. However, the calculations clearly show that the layered magnetic structures are considerably lower in energy as compared to a homogeneous magnetic ground state, *e*.*g*. 9.3 meV/f.u. The ordering of the tetrahedral chains considered only weakly influences the magnetic ground state (See Supplementary Note 2 and Figure S1), with parallel spins in the octahedral layers and antiparallel spins in the tetrahedral layers being the most stable. For STO strained ABO_2.5_, independent of the tetrahedral chain ordering, the most stable spin structure is ferromagnetic order in octahedral layers and G-type antiferromagnetic order in tetrahedral layers.

As shown by Mitra *et al*.^26^, the magnetic ground state can be explained by only including magnetic nearest neighbor interactions. For the system investigated, this means that the octahedrons will only interact with the other octahedrons within the same layer and the closest tetrahedral layers. Assuming that the coupling from one octahedral (tetrahedral) layer to another, through a tetrahedral (octahedral) layer, is low, the magnetic interaction between an octahedral layer and either G- or- C-like AFM ordered tetrahedral layer is similar in energy, as shown in Fig. 2. This can be rationalized from the spin structure, as both G- and C-type AFM tetrahedral layers have the same ratio of aligned/non-aligned spins adjacent to the octahedral layers, independent of the spin polarization direction in the octahedral layers. The magnetic coupling can depend on the Mn oxidation state. With the Sr doping investigated here, the average Mn oxidation state is +2.25 for the ABO_2.5_ system. Analyzing the magnetic moment of each site, we find that all of the tetrahedral sites have an oxidation state of +2, while half of the octahedral sites have a +2 state and the other half a +3 state. Using this to calculate the magnetic interaction parameters, *J*, we find that the least squares solution to the over-determined equation set based on the energies shown in Fig. 2 gives ferromagnetic coupling between two octahedrally coordinated Mn ions with *J*_*oct*−*oct*_ = 6.20 meV, and antiferromagnetic coupling between two tetrahedrally coordinated Mn ions with *J*_*tet*−*tet*_ = −3.49 meV. This is in agreement with two different magnetic sublattices in the octahedral and tetrahedral layers having parallel spins in each octahedral layer and anti-parallel spins in each tetrahedral layer.

#### ABO_2.67_ and ABO_2.75_

**Figure 3:**
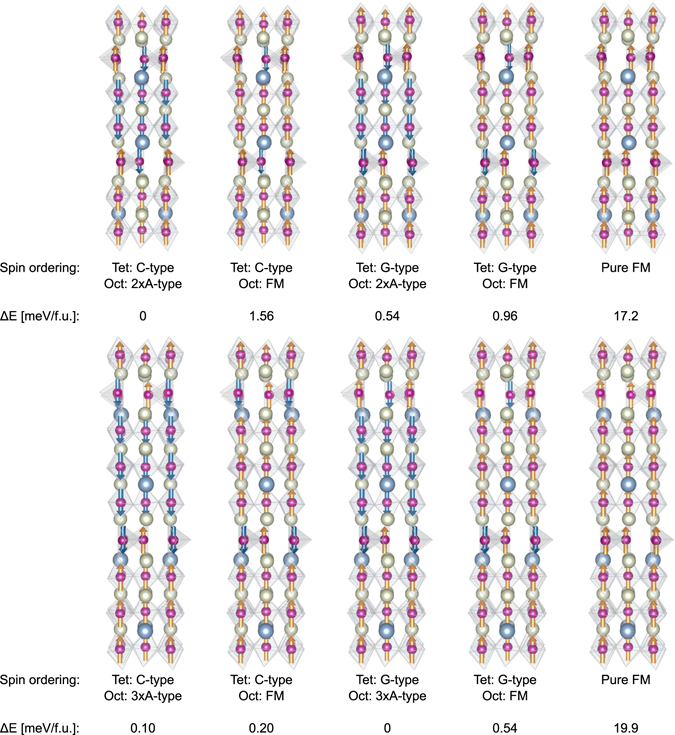
Energy difference of the low energy spin orderings, which all have an in-plane anti-parallel spins in the tetrahedral layers and in-plane parallel spins in the octahedral layers. Top: the ABO_2.67_ system, bottom: the ABO_2.75_ system. Pure ferromagnetic order is included as a reference.

Having established the magnetic ground state for the 1:1 ABO_2.5_, the effect of the oxygen stoichiometry is investigated by increasing *N*. Based on the results for *N* = 1 (ABO_2.5_) and *N* = ∞ (ABO_3_), we focus on the case with different magnetic sublattices in the octahedral and tetrahedral layers, where each octahedral layer have parallel spins and each tetrahedral layer have an antiferromagnetic ordering. The octahedral layers can all be polarized in the same direction giving a net moment; or every second or third octahedral layer can be polarized oppositely, called double or triple A-type for ABO_2.67_ and ABO_2.75_, respectively, resulting in no net moment. By the same arguments used for *N* = 1, C-type and G-type AFM ordering are possible for the tetrahedral layers, resulting in four different combinations of in-plane parallel spin ordering in the octahedral layers and in-plane anti-parallel spin orderings in the tetrahedral layers. As shown in Fig. 3, the energy differences between these possible spin configurations are small, both for the case of *N* = 2 (ABO_2.67_) and *N* = 3 (ABO_2.75_). Similar to the *N* = 1 (ABO_2.5_), any combination of parallel ordering in the octahedral layers and anti-parallel in the tetrahedral layers are considerably lower in energy than a pure FM state. For *N* = 2, ABO_2.67_, the lowest energy is found for the magnetic structure with double A-type octahedral layers and C-type tetrahedral layers, with an energy difference of 0.54 meV per formula unit compared to the next lowest energy spin ordering (Fig. 3). For *N* = 3, ABO_2.75_, the lowest energy is found for triple A-type octahedral layers and G-type ordered tetrahedral layers. Also for the *N* = 2, ABO_2.67_ there is only a weak dependence of the spin ordering to the tetrahedral chain type, see Supplementary Note 2 and Figure S2.

### Electronic structure

**Figure 4:**
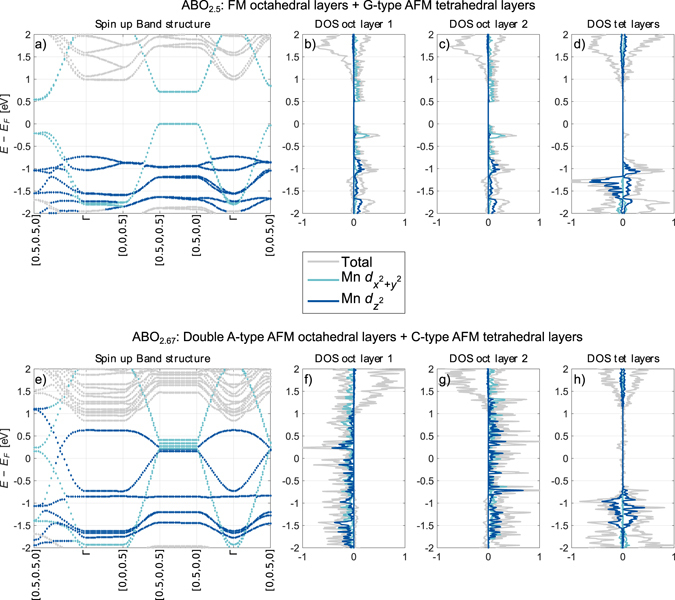
Band structure and layer resolved density of states (DOS) for the octahedral and tetrahedral layers of LSMO brownmillerite (**a**–**d**) ABO_2.5_ with ferromagnetic coupling in the octahedral layers and G-type antiferromagnetism in the tetrahedral layers, and (**e**–**h**) ABO_2.67_ with A-type antiferromagnetism in the octahedral layers and C-type antiferromagnetism in the tetrahedral layers. For the bands, the *k*_*x*_ and *k*_*y*_ *k*_*y*_ are parallel to the layers, while the *k*_*z*_ is normal to the layers. The bands and DOS are further projected onto spherical harmonics. The bands where the projection on the Mn: ${d}_{{x}^{2}-{y}^{2}}$ and Mn: ${d}_{{z}^{2}}$ orbitals on each k-point exceeds 33% are coloured according to the legend; the rest of the points are coloured grey. (**b**,**c**), and (**f**,**g**) show the projection on the different octahedral layers for ABO_2.5_ and ABO_2.67_ respectively (DOS oct layer), while (**d**) and (**h**) shows the sum of both tetrahedral layers (DOS tet layer).

Next, the electronic properties are correlated with the orbital ordering to rationalize how the magnetic structure evolves with *N*, the number of octahedral layers between each tetrahedral layer. The spin up band structure and layer resolved electronic density of states (DOS) are shown in Fig. 4, only the band structure for the spin configurations with the lowest energy of ABO_2.5_ and ABO_2.67_ are shown, as the band structure of ABO_2.75_ is qualitatively similar to that of ABO_2.67_. Further, the only notable difference between the different spin orderings is whether the two different octahedral layers are polarized parallel or anti-parallel. The band structures and DOSes for spin down and ABO_2.75_ are shown in Supplementary Note 3. When inspecting the layer resolved DOSes we see that the tetrahedral layers have a band gap of ~1.5 eV for all magnetic structures. The octahedral layers display either an indirect band gap of ~0.5 eV for ABO_2.5_, or no band gap (metallic) for ABO_2.67_ and ABO_2.75_. Furthermore, the band structure has low dispersion perpendicular to the layers compared to the in-plane band directions. Such flat bands perpendicular to the layers imply large effective mass and low electronic mobility out-of-plane compared to in-plane, resulting in strongly anisotropic electronic conductivity^11, 29^. With a large band gap only in the tetrahedral layers as well as low dispersion perpendicular to the layers, spin polarized two-dimensional electronic conduction in the octahedral layers is possible: a quasi-2D spin polarized electron gas. We note that, as the top of the conduction band and the bottom of the valence band consists mainly of octahedral states, there are only minute differences in the electronic structure around the Fermi level for different tetrahedral chain orderings.

**Figure 5:**
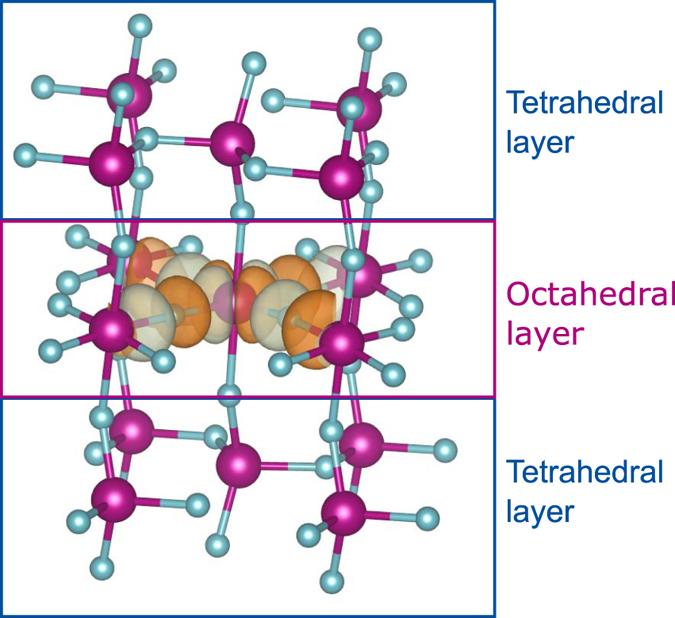
Iso-surface plot of the maximally localized Wannier functions for the valence band of the LSMO ABO_2.5_ structure centered on octahedrally coordinated Mn. Positive isosurfaces are coloured orange while negative isosurfaces are green. The iso-surface level is set to 1.

In order to illustrate the 2D localization of the charge carriers maximally localized Wannier functions^30, 31^ of the valence band and conduction band of the ABO_2.5_ structure are calculated. An iso-surface plot of the maximally localized Wannier function is showed in Fig. 5. The Wannier function has a ${d}_{{x}^{2}-{y}^{2}}$ character and is confined to the octahedral layers. Based on the Wannier function and the projected DOS, the Goodenough-Kanamori rules^32, 33^ can explain the spin ordering within in-plane ferromagnetically coupled octahedral sites. With the current Sr doping, half of the octahedral sites have a *d* ^*5*^ (Mn^2+^) state and the other half a *d* ^*4*^ (Mn^3+^) state. Hence the direction of the e_g_ orbitals then determines if the interaction is AFM superexchange or FM double exchange. As seen in Figs 4 and 5, the ${d}_{{x}^{2}-{y}^{2}}$ states from Mn^2+^ are at the top of the valence band, pointing towards the Mn^3+^ sites which are mainly occupied by ${d}_{{z}^{2}-r}$ states. Hence, the magnetic coupling between the octahedral layers should be in-plane double exchange and ferromagnetic according to the Goodenough-Kanamori rules. When the number of octahedral layers between each tetrahedral layer increases, an increased mix of ${d}_{{x}^{2}-{y}^{2}}\,\,$ and ${d}_{{z}^{2}-r}$ states at the Fermi level give rise to strong double exchange in each octahedral layer and a metallic state. We note, however, that the Goodenough-Kanamori rules can not be used to determine the exchange type for tetrahedral sites^33^.

### Strain dependence

**Figure 6:**
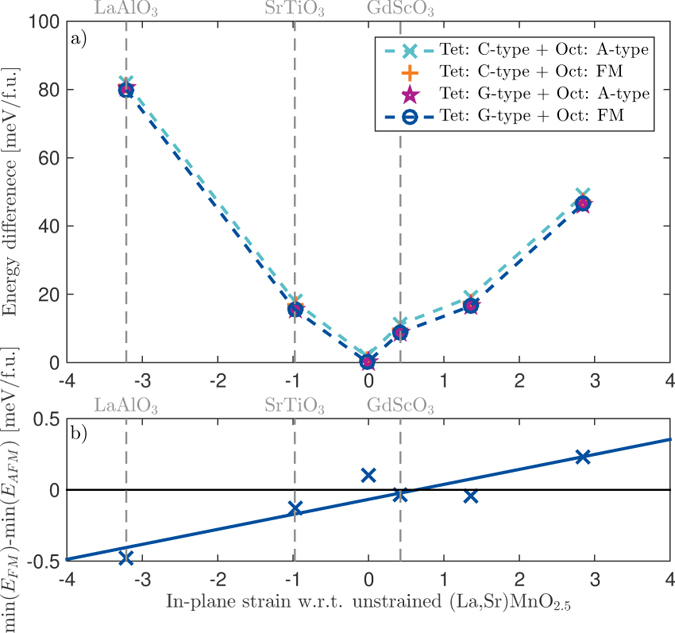
(**a**) Energy difference as a function of in-plane strain for the four low energy magnetic structures. Note that the evolution around the zero strain point deviates from the expected parabolic shape because here the in-plane lattice vectors are no longer equal. The dashed coloured lines are guides to the eye, while the grey vertical lines represent the strain from some commercially available substrates. (**b**) Energy difference between the lowest ferromagnetic structure and the lowest antiferromagnetic structure in (La, Sr) MnO_2.5_ brownmillerite as a function of in-plane strain. Positive values mean that the structures where the octahedrons are A-type antiferromagnetic (AFM) coupled have the lowest energy, while negative values means that ferromagnetic (FM) coupling of the octahedrons are favored, the solid line correspond to a linear fit to the data.

Finally, we turn to the possibility to tune the magnetic ground state through epitaxial strain for the case of one octahedral layer between every tetrahedral layer (ABO_2.5_). The energy difference between FM and A-type octahedral layers has an almost linear dependence on in-plane strain, as shown in Fig. 6. The approximate 1% compressive strain resulting from preparing ABO_2.5_ (LSMO) on a SrTiO_3_ substrate is just on the side where a FM ordering of the octahedral layers is calculated to have slightly lower energy than an AFM ordering of the octahedral layers. However, the energy differences between the different spin states are small and within the uncertainty of the calculations. These small energy differences can also point towards high magnetic susceptibility due to multiple close lying transition temperatures^34^. For increased compressive strain, *e*.*g*. from a LaAlO_3_ substrate, the FM ordering is more favored, with an energy difference of approximately 0.47 meV per formula unit. If a tensile strain is applied, *e*.*g*. approximately 3%, AFM ordering of the octahedral layers is favored, with an energy difference of 0.23 meV per formula unit, compared to the lowest energy FM coupling between the octahedral layers. The energy difference between FM and AFM coupled octahedral layers shows a linear positive response to in-plane strain (Fig. 6b), indicating that antiferromagnetic coupling from one octahedral layer to another increases when the out-of-plane lattice parameter decreases. These results points to the possibility of using strain to engineer also the magnetic properties in materials with ordered oxygen vacancies^35–37^.

## Conclusion

Layered ordering of vacancies in anion deficient perovskite structures is a novel route to induce anisotropic properties similar to what can be achieved with cation vacancies in the Ruddlesden-Popper, Aurivillius and Dion-Jacobsen families. For LSMO we show that these oxygen deficient systems are prone to show an in-plane spin coupling that is dominating the out-of-plane coupling. The in-plane coupling in the octahedral layers is shown to be ferromagnetic, while for the tetrahedral layers the in-plane coupling is antiferromagnetic. Different combinations of in-plane FM octahedral layers and AFM tetrahedral layers are close in energy and should thus be tunable through external means such as epitaxial strain, chemical doping or applied magnetic field. As the band gap in the tetrahedral layers is 1.5 eV, and the system exhibit flat bands perpendicular to the layering direction, a confined spin polarized conduction can be foreseen in the octahedral layers, where the band gap is zero with significant band dispersion along in-plane directions. These results point to the possibility to rely on anion ordering to control functional properties for device applications.

## Methods

The DFT calculations were performed with the Projector Augmented Wave (PAW)^38, 39^ method as implemented in the Vienna Ab-initio Simulation Package (VASP)^39, 40^, with the Perdew-Burke-Ernzerhof generalized gradient approximation for solids (PBEsol)^41^. The plane wave cutoff energy was set to 550 eV and PAW-potentials with 11, 10, 15 and 6 valence electrons were used for La, Sr, Mn and O, respectively. GGA + U (Dudarev *et al*.^42^) was used with U = 3 eV for Mn 3d and U = 10 eV for La 4 f states^17, 43, 44^. For stoichiometric LSMO we used a 40 atom La_6_Sr_2_Mn_8_O_24_ cell with a 4 × 4 × 4 Γ-centered k-point mesh to sample the Brilluion zone, with corresponding k-point densities for oxygen deficient cells. The Sr atoms were distributed evenly across the unit cell, and different possible distributions were tested. Biaxial strain was simulated by fixing the in-plane lattice parameter to those calculated for the respective substrates, while the out of plane lattice parameter was allowed to relax. Atomic positions and lattice vectors were relaxed until the Hellmann-Feynman forces on the ions were below 0.01 eV/Å. Maximally localized Wannier functions were calculated from the DFT-Bloch functions with the wannier90 code^30, 31, 45^. For a more detailed description of the calculation details, please see Supplementary Note 1.

## Electronic supplementary material

Supplementary Note
