# Supplementary material for: Spatially Confined Spin Polarization and magnetic sublattice control in (La,Sr)MnO3−δ Thin Films by Oxygen Vacancy Ordering
Source: Sci Rep. 2017 Jun 29;7:4386. doi: 10.1038/s41598-017-04103-y (PMC5491515; doi:10.1038/s41598-017-04103-y)
Supplement: Supplementary file 1 — Supplementary Note [file 41598_2017_4103_MOESM1_ESM.doc]

Spatially Confined Spin Polarization and magnetic sublattice control in (La,Sr)MnO_3-δ_ Thin Films by Oxygen Vacancy Ordering

Magnus Moreau,^1^ Sverre M. Selbach,^2^ and Thomas Tybell^1^,*

1. Department of Electronics and Telecommunications, NTNU, 7491 Trondheim, Norway
2. Department of Materials Science and Engineering, NTNU, 7491 Trondheim, Norway

*) Corresponding Author

**Supplementary Note 1: Computational details**

The DFT calculations were performed with the Projector Augmented Wave (PAW) method as implemented in the Vienna Ab-initio Simulation Package (VASP),[^3-6^](#_ENREF_3) with the Perdew-Burke-Ernzerhof generalized gradient approximation for solids (PBEsol).[^7^](#_ENREF_7) The PAW-potentials treating 11 valence electrons for La (4*s*^2^4*p*^6^5*d*^1^6*s*^2^), 10 for Sr (4*s*^2^4*p*^6^5*s*^2^), 15 for Mn (3*s*^2^3*p*^6^3*d*^5^4*s*^2^) and 6 for O (2*s*^2^2*p*^4^) supplied with VASP were used. We use the rotational invariant GGA+U approach introduced by Dudarev et al.[^8^](#_ENREF_8) Tests for different U values were done in the range of 0-5 eV for the Mn 3d electrons, and a value of 3 eV was chosen as this well reproduces the density of states of stoichiometric bulk LSMO, this value has also previously well described manganese oxides with oxygen vacancies. Tests for a Hubbard U values in the range 0-12 eV were performed for the La 4f orbitals, and a value of 10 eV was chosen in order to move the 4f states away from the Fermi level[^11^](#_ENREF_11). A cutoff energy of 550 eV was used for the plane wave basis set. For stoichiometric LSMO a 40 atoms La_6_Sr_2_Mn_8_O_24_ was used with a 4x4x4 Γ-centered k-point mesh to sample the Brilluion zone. A 36 atom La_6_Sr_2_Mn_8_O_20_ unit cell with a 6x6x2 Γ-centered k-point mesh for Brillouin zone was used for calculation of the oxygen deficient structures with general formula ABO_2.5_. For the structures with general formula ABO_2.67_ and ABO_2.75_ a k-point mesh of 6x6x1 points centered on the
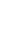
 point was used, while the unit cell consisted of La_9_Sr_3_Mn_12_O_32_ and La_12_Sr_4_Mn_16_O_44_ respectively. Different distributions of the Sr atoms were tested, however it did not influence the relative energies of the magnetic orderings, unless all the Sr atoms were clustered in one corner of the unit cell. To accommodate for the Pbcm-like symmetry with Left-Right Right-Left (LR-RL) tetrahedral chain order, the cell was doubled along the in-plane b-axis and the number of K-points along this direction was halved. To simulate the epitaxial strain of a substrate, the in plane lattice parameter was fixed in accordance with the equilibrium lattice parameter calculated for the respective substrate, while the out of plane lattice parameter was allowed to relax. The atomic positions and lattice vectors were relaxed until the Hellmann-Feynman forces on the ions were below 0.01 eV/Å. Collinear spins on the Mn ions were assumed for all calculations. These parameters reproduced the experimentally known ferromagnetic ground state of LSMO when strained to a STO substrate (calculated lattice parameter for cubic STO: 3.896 Å) with a energy difference of 42 meV compared to the lowest antiferromagntic ordering (see supplementary info). The maximally localized Wannier functions were calculated from the DFT-Bloch functions with the wannier90 code.[^12-14^](#_ENREF_12)

**Supplementary Note 2: Tetrahedral chain order**

As discussed in detail by Young and Rondinelli,[^15^](#_ENREF_15) the tetrahedral chains can be ordered in three symmetry inequivalent patterns depending on the chirality of the tetrahedral chain rotations. These rotation patterns are left-left left-left (LL-LL) with a *I*2*bm*-like symmetry, right-left left-right RL-LR with *Pbcm*-like symmetry or left-left right-right (LL-RR) with *Pnma*-like symmetry. As shown in Figure S1, for the SrTiO_3_ strained ABO_2.5_ system the RL-LR *Pbcm*-like symmetry has the lowest energy,
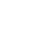
5 meV with respect to the LL-LL *I*2*bm*-like symmetry. However, different tetrahedral chains give an energy cost that is almost independent of the spin ordering. For a given magnetic ordering, the RL-LR tetrahedral chain is always lowest in energy followed by LL-LL ordering and finally the LL-RR, the only exception to this is seen for the C-type ordering, which is high in energy and thus not important for the physical properties. For the
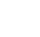
 ABO_2.67_ a similar development of the tetrahedral chain ordering is found, the lowest energy is for the LL-LL tetrahedral chain pattern. Here we also see that the four different laying magnetic orderings with parallel spins in octahedral layers and anti-parallel spins in the tetrahedral layers, are similar in energy for a given tetrahedral chain pattern.


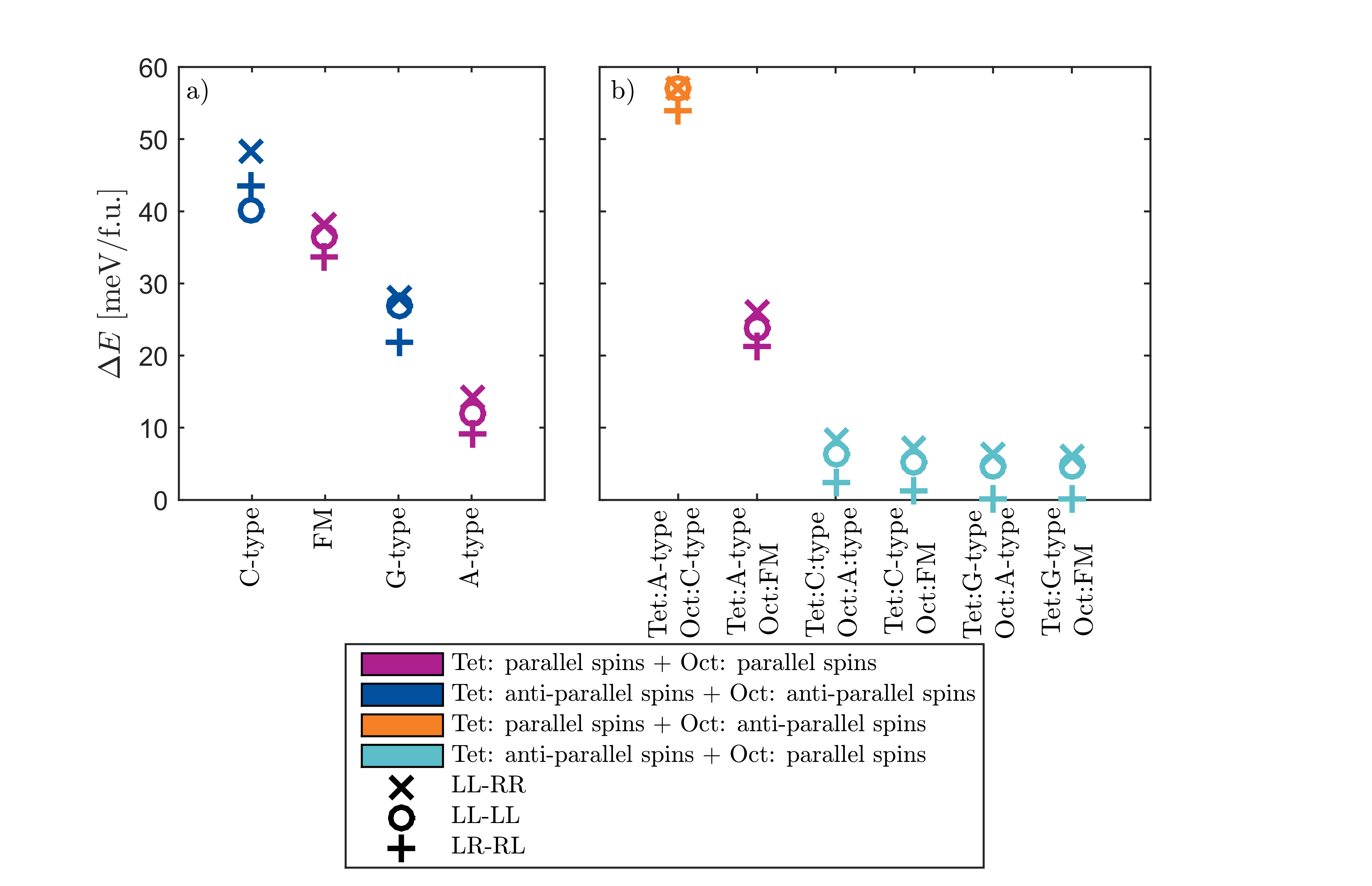
**Figure S1**, Energy difference between the different spin orderings and tetrahedral chains for the SrTiO_3_-strained ABO_2.5_ system.
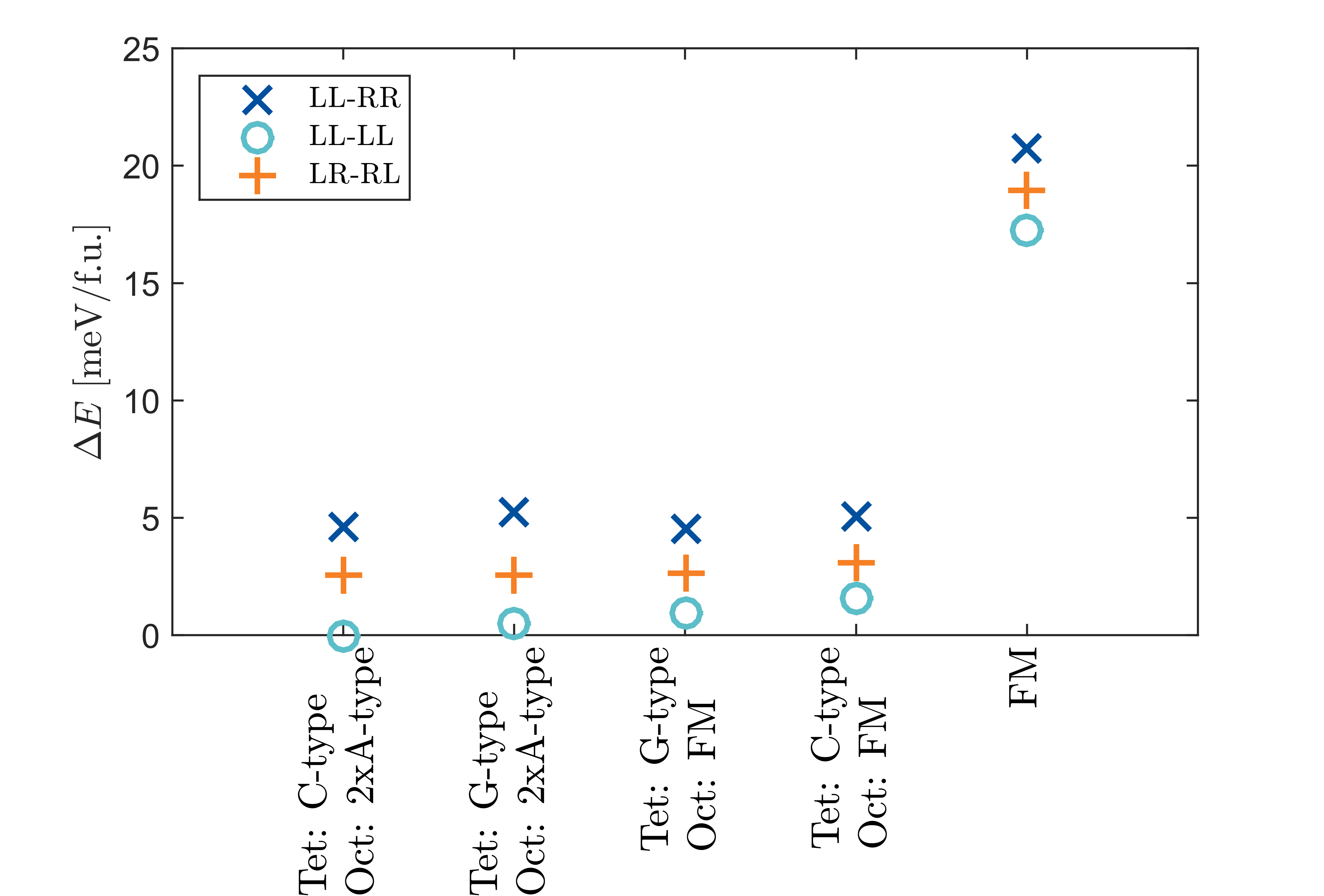


**Figure S2,** energy difference between the four different low laying magnetic orderings with parallel spins in octahedral layers and anti-parallel spins in the tetrahedral layers, and compared to the pure ferromagnetic structure.

**Supplementary Note 3: Electronic structure**

Figure S3 – S5 show the density of states for the four different low energy spin orderings as a function of oxygen stoichiometry. No qualitative change is observed when going from ABO_2.67_ to ABO_2.75_ as seen in Figure S4 and S5. Further, as shown in figure S5, there are also only small differences between the ferromagnetically coupled octahedral layers in the ABO_2.75_ and ABO_3_ system, indicating that 2-3 layers is enough to get bulk like properties between the tetrahedral layers.

Figure S6 and S7 show the band structure for the spin up and spin down bands respectively for the four different low energy spin orderings as a function of oxygen stoichiometry. The figures show that the in-plane confinement of carriers are present for all spin orderings with in-plane parallel spins in the octahedral layers and in-plane antiparallel spins in the tetrahedral layers.


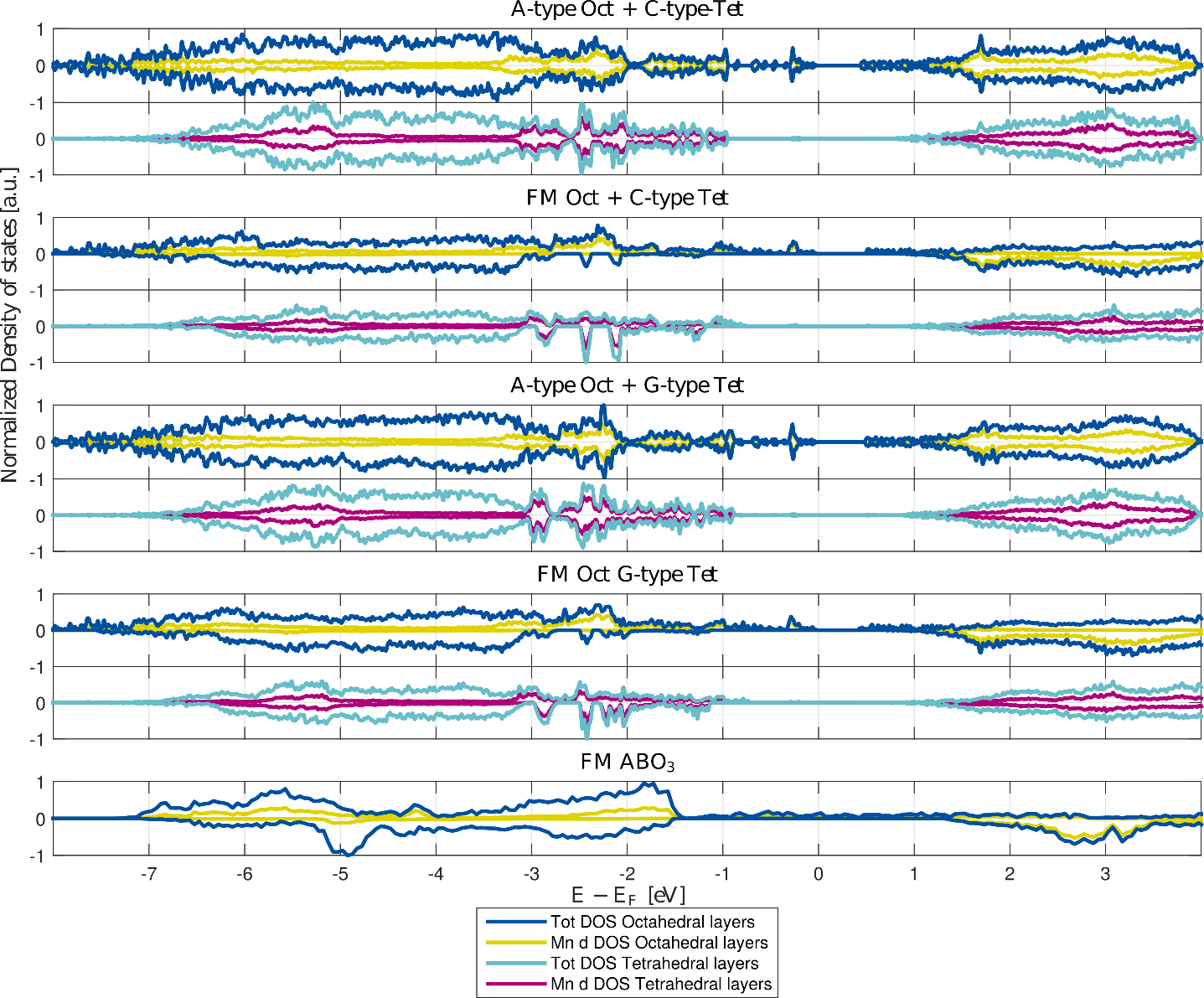


**Figure S3,** DOS for the different low energy spin orderings for ABO_2.5_, ABO_3_ included as a reference. Note that the two octahedral layers are summed up to one, unlike what is shown in Figure 4 in the main paper.


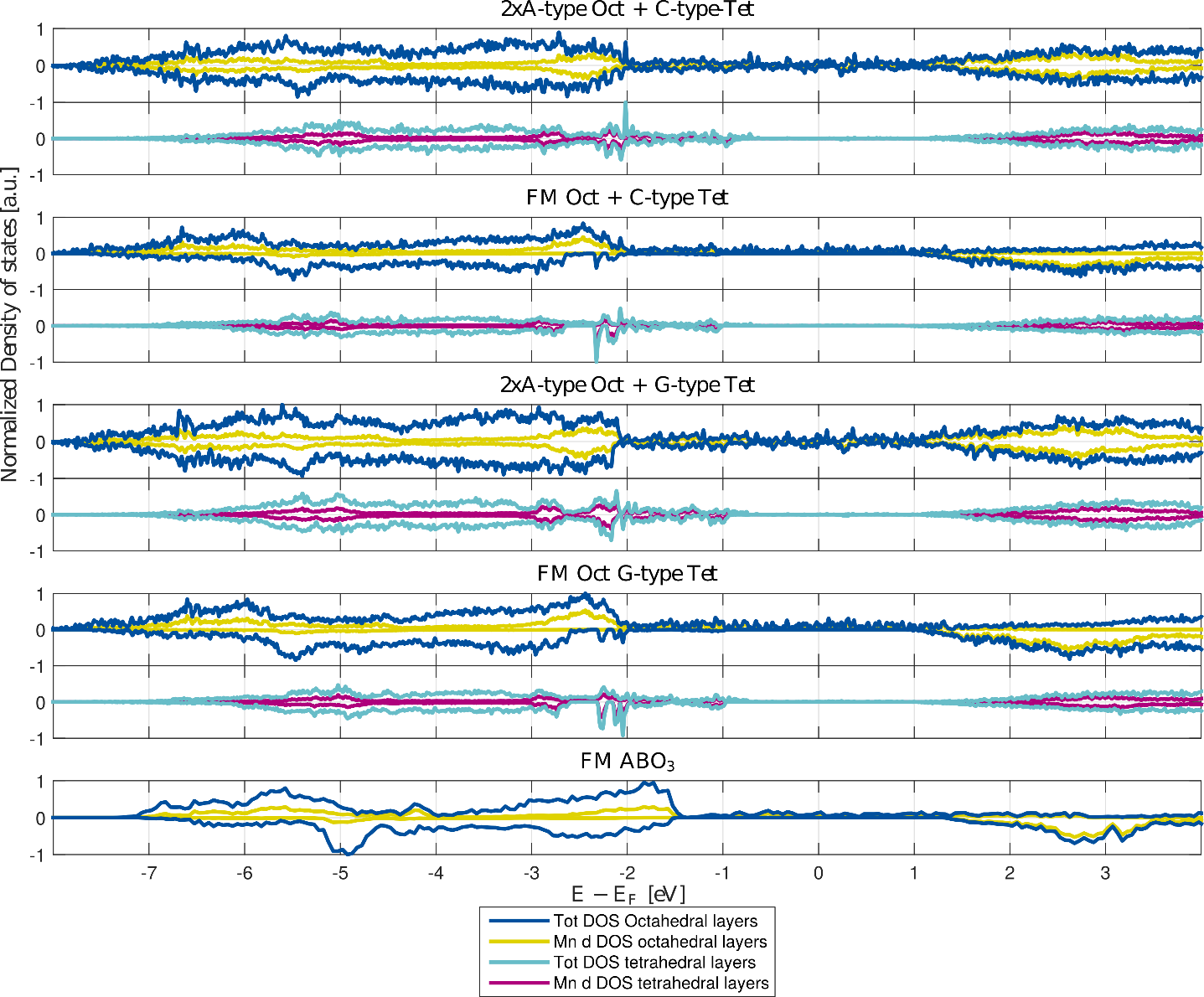
**Figure S4,** DOS for the different low energy spin orderings for ABO_2.67_, ABO_3_ included as a reference. Note that the two octahedral layers are summed up to one, unlike what is shown in Figure 4 in the main paper.


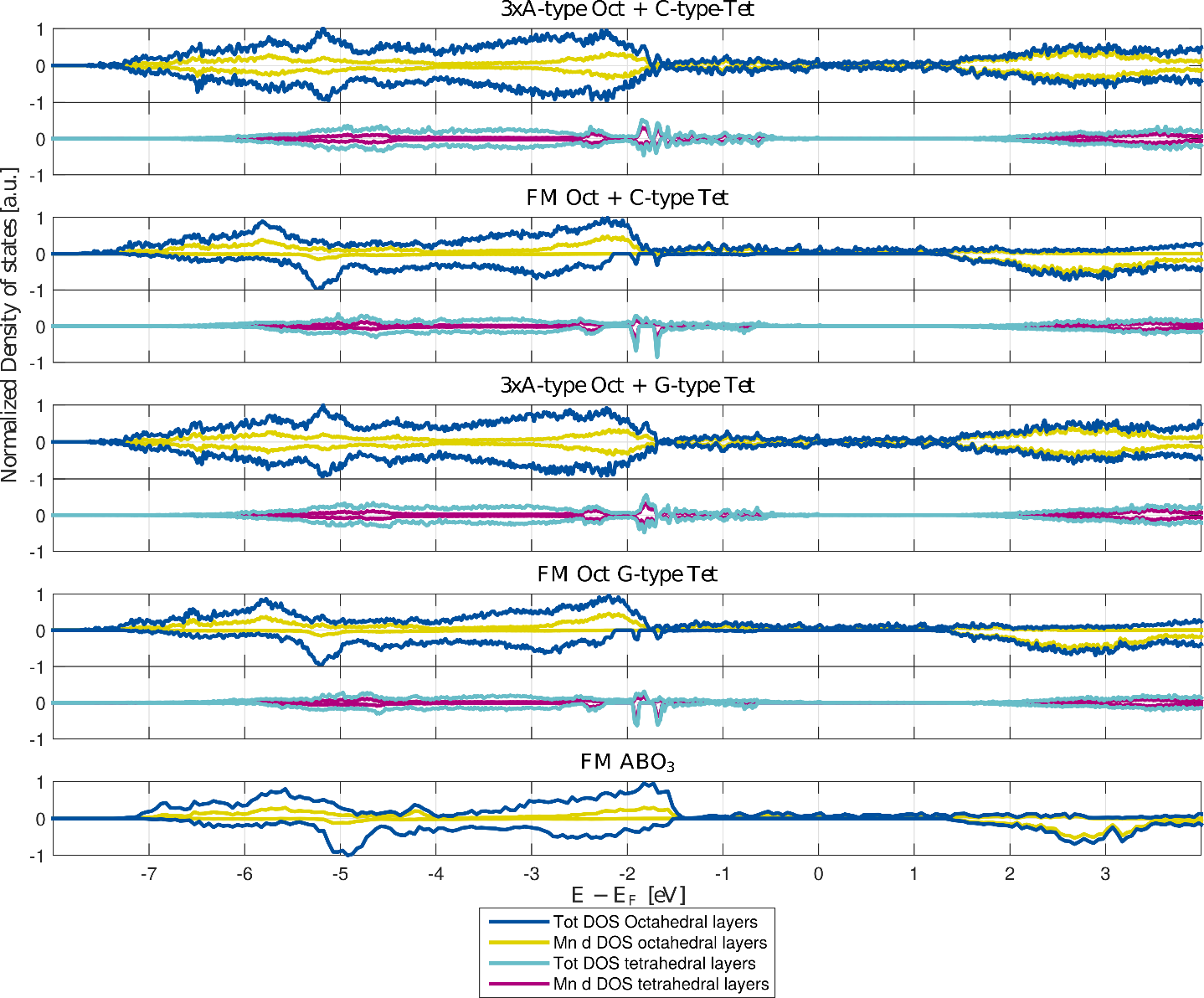


**Figure S5,** DOS for the different low energy spin orderings for ABO_2.75_, ABO_3_ included as a reference. Note that the two octahedral layers are summed up to one, unlike what is shown in Figure 4 in the main paper.


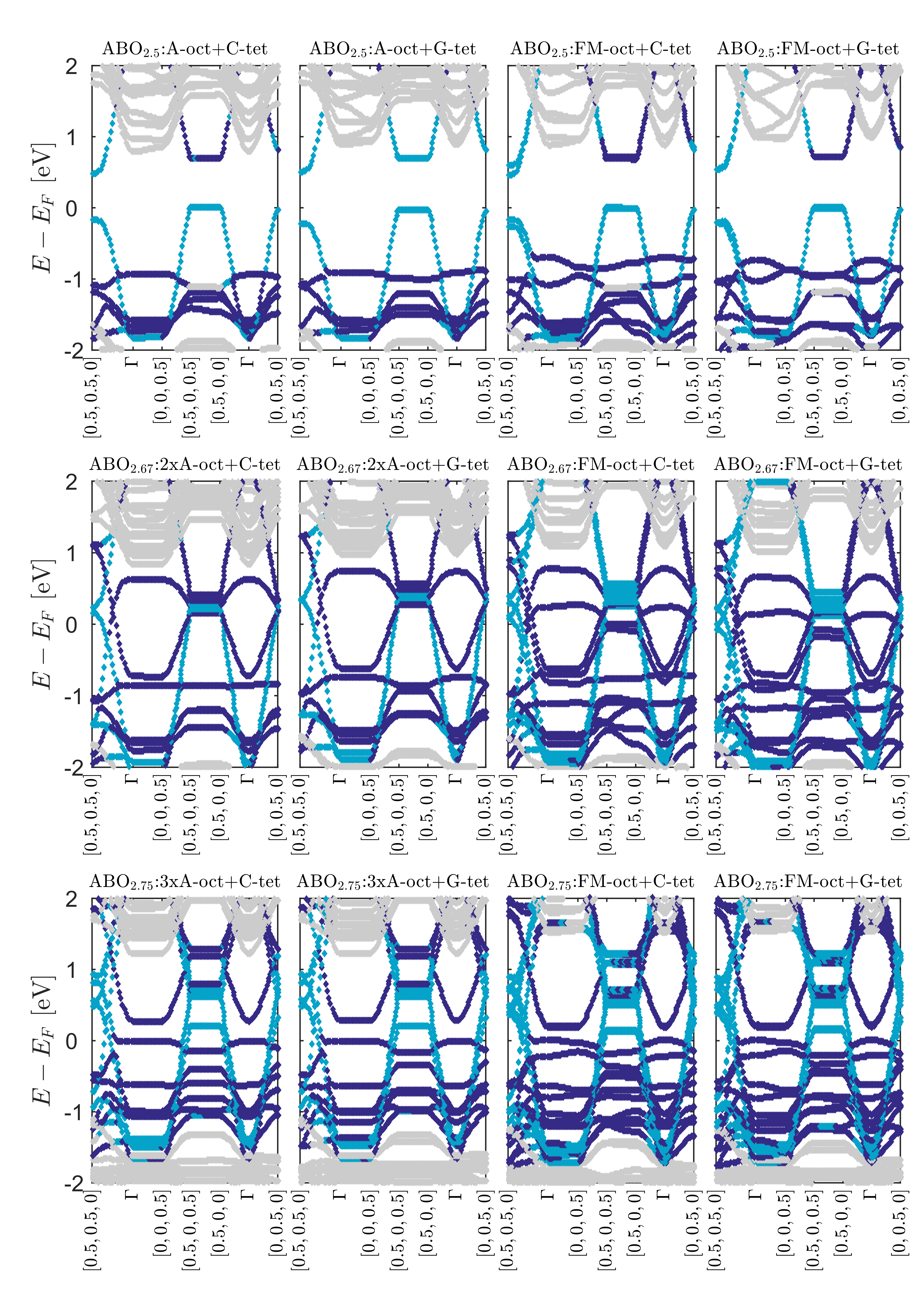


**Figure S6,** Spin up band structure for the different low energy spin orderings as a function of oxygen stoicometry.

**
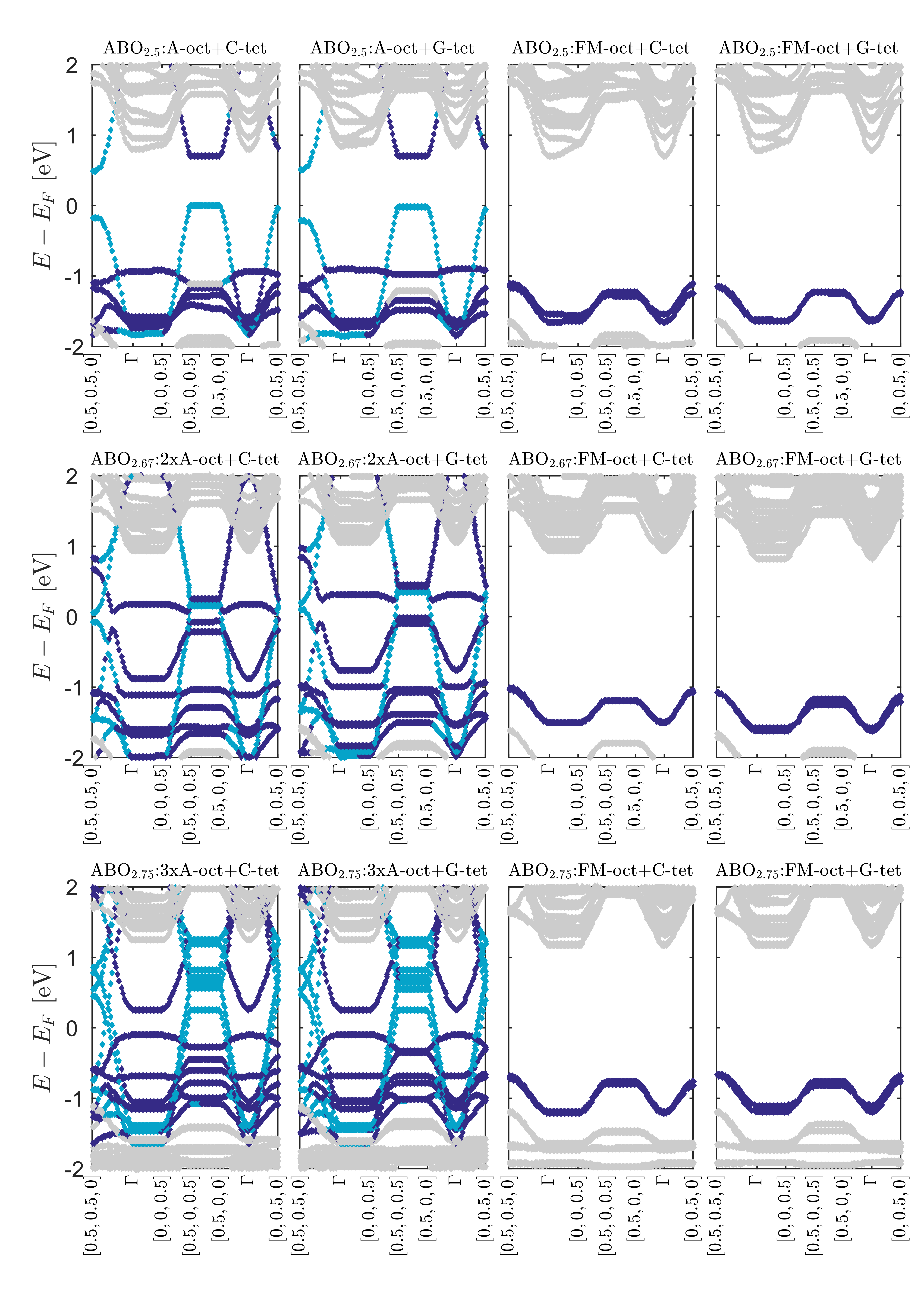
Figure S7,** Spin down band structure for the different low energy spin orderings as a function of oxygen stoicometry

**References**

1 Blochl, P. E. Projector Augmented-Wave Method. *Phys. Rev. B* **50**, 17953-17979, (1994).

2 Kresse, G. & Joubert, D. From Ultrasoft Pseudopotentials to the Projector Augmented-Wave Method. *Phys. Rev. B* **59**, 1758-1775, (1999).

3 Kresse, G. & Furthmuller, J. Efficiency of ab-initio total energy calculations for metals and semiconductors using a plane-wave basis set. *Comput. Mater. Sci.* **6**, 15-50, (1996).

4 Kresse, G. & Furthmuller, J. Efficient Iterative Schemes for Ab Initio Total-Energy Calculations Using a Plane-Wave Basis Set. *Phys. Rev. B* **54**, 11169-11186, (1996).

5 Kresse, G. & Hafner, J. Abinitio molecular-dynamics for liquid-metals *Phys. Rev. B* **47**, 558-561, (1993).

6 Kresse, G. & Hafner, J. Ab-initio molecular-dynamics simulation of the liquid-metal amorphous-semiconductor transition in germanium *Phys. Rev. B* **49**, 14251-14269, (1994).

7 Perdew, J. P. *et al.* Restoring the Density-Gradient Expansion for Exchange in Solids and Surfaces. *Phys. Rev. Lett.* **100**, 136406, (2008).

8 Dudarev, S. L., Botton, G. A., Savrasov, S. Y., Humphreys, C. J. & Sutton, A. P. Electron-Energy-Loss Spectra and the Structural Stability of Nickel Oxide: An LSDA+U Study. *Phys. Rev. B* **57**, 1505-1509, (1998).

9 Aschauer, U., Pfenninger, R., Selbach, S. M., Grande, T. & Spaldin, N. A. Strain-Controlled Oxygen Vacancy Formation and Ordering in CaMnO_3_. *Phys. Rev. B* **88**, 054111, (2013).

10 Pavone, M., Muñoz-García, A. B., Ritzmann, A. M. & Carter, E. A. First-Principles Study of Lanthanum Strontium Manganite: Insights into Electronic Structure and Oxygen Vacancy Formation. *J. Phys. Chem. C* **118**, 13346-13356, (2014).

11 Johnson-Wilke, R. L. *et al.* Quantification of Octahedral Rotations in Strained LaAlO_3_ Films via Synchrotron X-ray Diffraction. *Phys. Rev. B* **88**, 174101, (2013).

12 Souza, I., Marzari, N. & Vanderbilt, D. Maximally Localized Wannier Functions for Entangled Energy Bands. *Phys. Rev. B* **65**, 035109, (2001).

13 Mostofi, A. A. *et al.* wannier90: A Tool for Obtaining Maximally-Localised Wannier Functions. *Comput. Phys. Commun.* **178**, 685-699, (2008).

14 Marzari, N. & Vanderbilt, D. Maximally Localized Generalized Wannier Functions for Composite Energy Bands. *Phys. Rev. B* **56**, 12847-12865, (1997).

15 Young, J. & Rondinelli, J. M. Crystal structure and electronic properties of bulk and thin film brownmillerite oxides. *Phys. Rev. B* **92**, 174111, (2015).

16 Momma, K. & Izumi, F. VESTA 3 for Three-Dimensional Visualization of Crystal, Volumetric and Morphology Data. *J. Appl. Crystallogr.* **44**, 1272-1276, (2011).
